# The Digital Exposome: A Life Course Framework for Health in the Digital Age

**DOI:** 10.2196/90153

**Published:** 2026-05-08

**Authors:** Pascal Petit, Nicolas Vuillerme

**Affiliations:** 1 Univ. Grenoble Aples, CNRS, Grenoble INP, LIG, SANGRIA, Grenoble, 38000, France; 2 Institut Universitaire de France, Paris, France

**Keywords:** digital health, life course health, digital exposome, digital determinants of health, health equity, digital public health, digital technologies, mobile phone, digital phenotype, wearables

## Abstract

Digital technologies are reshaping human behavior, health care delivery, and population health; however, their cumulative effects across the lifespan remain underexplored. This viewpoint argues that exposures arising from interactions with digital technologies should be formally integrated into exposome science as a distinct, measurable component of the human environment. Our aims are to (1) redefine the digital component of the exposome (the digital exposome) within the broader exposome framework, (2) examine its life course implications for health and equity, and (3) outline a research and policy agenda to enable its systematic measurement and integration into clinical and public health practice. Digital technology–related exposures can confer benefits such as enhanced health monitoring, personalized interventions, improved access to care, and the promotion of healthy behaviors. However, they may also introduce potential risks, including mental health challenges, cognitive and circadian disruptions, sedentary lifestyles, exposure to misinformation, and widening inequities among vulnerable populations. Despite their ubiquity, digital technology–related exposures remain poorly integrated into clinical medicine, epidemiology, or public and global health policies. Drawing on interdisciplinary evidence from exposure science, epidemiology, and digital phenotyping research, we propose a refined conceptual definition of the digital exposome grounded in the classical exposome domains. We propose redefining the digital exposome as the full spectrum of exposures resulting from interactions or proximities with digital technologies and their combined influence on health across the lifespan. This framework conceptualizes digital technology–related exposures as a dynamic set of environmental influences operating through sociotechnical, behavioral, and biological pathways over the life course. To operationalize this framework, we discuss practical approaches using validated behavioral instruments, objective device use logs, ecological momentary assessments, smartphone-based digital phenotyping, and wearable sensing technologies. Systematic measurement, large-scale longitudinal studies, and harmonized exposure metrics are needed to characterize the cumulative health impacts of digital environments more accurately. Emerging tools such as digital markers or biomarkers and digital phenotypes offer promising opportunities to link real-world technology use with physiological and biological outcomes, thereby supporting precision medicine and population health strategies. Ethical governance, privacy safeguards, and equity considerations must be embedded from the start, drawing on emerging exposomethics frameworks. Recognizing the digital exposome as a modifiable determinant of health offers a foundation for evidence-based guidance, prevention strategies, and policy interventions suited to increasingly digital societies. By integrating digital technology–related exposures into exposome science, clinical practice, and public health research, this viewpoint seeks to foster interdisciplinary dialogue, guide future empirical work, and support the development of safer and more equitable digital environments across the lifespan.

## A Constantly Evolving Digital World

Societies worldwide are undergoing a profound digital transformation, as information and communication technologies increasingly shape everyday life and influence health and well-being across the lifespan [1-3]. From early childhood to older age, digital technologies (DTs) influence education, health care delivery, communication, and social connection, fundamentally reshaping how people live, learn, and interact [1,4]. Individuals now navigate environments characterized by rapid connectivity, omnipresent media platforms, and instantaneous access to health-related information [1-3].

DTs have become powerful enablers of health and well-being, supporting the broader vision of health for all [5]. Artificial intelligence (AI)-driven tools, wearable devices, mobile health, and eHealth technologies (eg, telemedicine platforms) facilitate exposure and health monitoring, improve access to care, and support personalized interventions [4-8]. In digital psychiatry, for instance, passive data streams and AI-based tools are increasingly used to infer mental health states and support personalized care [8,9]. Educational technologies can also foster learning and cognitive development [4].

However, the influence of DTs on health is inherently dual. While they offer substantial benefits, they also introduce potential risks, particularly for vulnerable populations such as children, older adults, and individuals facing barriers related to digital literacy, infrastructure, or socioeconomic conditions [3,4,10,11]. For example, some forms of digital media use, such as exchanging SMS text messages with close friends or viewing positive or nature-related images, have been associated with improvements in well-being [12]. In contrast, prolonged screen time or exposure to artificial light at night has been linked to adverse health outcomes [12].

The emerging concept of the digital determinants of health highlights how technological factors influence disparities in access, affordability, and health outcomes [13,14]. These determinants interact dynamically with social determinants of health (SDOH), often exacerbating inequalities and influencing quality of care and accessibility [13,14]. Recognizing these interactions is essential for achieving equitable health progress in increasingly digital societies. The dual nature of digital influence underscores the need for conceptual frameworks that address both the benefits and the emerging risks of digital transformation.

In this context, the exposome concept [15,16] provides a promising framework for understanding the cumulative impact of digital environments on health. This viewpoint develops a conceptual and operational framework for integrating DT-related exposures into the exposome paradigm. Our primary aims are to (1) redefine the digital exposome within the broader exposome framework, (2) articulate its implications for health and equity across the life course, and (3) outline a research and policy agenda to support its measurement and integration into clinical and public health practice.

The intended audience includes digital health researchers, exposome scientists, clinicians, public health professionals, policymakers, sociologists, and ethics scholars. By bridging these disciplines, this viewpoint seeks to translate the concept of the digital exposome into actionable research agendas, measurement strategies, and policy-relevant interventions. It aims to stimulate interdisciplinary dialogue; guide future empirical research (eg, digital phenotyping and digital marker or biomarker development); and inform decision-making in clinical, public health, digital health, and policy contexts.

A targeted narrative literature review informed this viewpoint by identifying relevant publications from databases such as PubMed, Web of Science, Dimensions, and Google Scholar, using search terms related to the exposome, DT, and health.

## Mapping Digital-Related Exposures

The exposome concept, representing the totality of environmental influences from conception to death [15,16], offers a powerful paradigm for characterizing the full spectrum of DT-related exposures across the life course. However, despite their ubiquity in modern societies, DT-related exposures remain largely absent from current exposome frameworks [1,2]. Only a limited number of studies have begun to explore their integration into exposomic research [17,18].

Recent investigations illustrate both the feasibility and potential value of incorporating DT-related exposures into exposome science. For instance, a data-driven exposome-wide association study (ExWAS) introduced digital exposomic risk scores that quantify cumulative exposure to digital risks such as general usage, cyberbullying, and problematic or addictive behavior in adolescents [17]. These scores explained a substantial proportion of the variance in self-reported mental health and enhanced understanding of how DT-related exposures contribute to mental health inequities [17]. Similarly, machine learning analyses of UK Biobank data examined dementia risk in relation to 128 exposome factors, including DT-related exposures, and found an inverse association between dementia and the duration of mobile phone use [18]. Although causal inferences remain uncertain, these findings highlight the potential relevance of DT-related exposures within broader environmental health research.

These pioneering studies highlight the necessity, feasibility, and potential utility of integrating DT-related exposures into exposomic research at both the population and individual levels. As individuals spend increasing portions of their lives online or connected digitally, these exposures must be recognized as meaningful components of the exposome [1]. Consequently, the question is no longer whether DT-related exposures belong within exposome science, but rather how they can be systematically measured, modeled, and integrated. Expanding the exposome framework to encompass DT-related exposures may help bridge critical knowledge gaps and support the development of patient-centered, evidence-based interventions in an increasingly digitalized world.

## Redefining the Digital Exposome

Previous efforts have defined the digital exposome as “the whole set of tools and platforms (including contents) that an individual uses and the activities and processes that an individual engages with as part of his digital life” [1]. While foundational, this definition remains too narrow to capture the full complexity of lifelong DT-related exposures.

We propose redefining the digital exposome as the complete and cumulative collection of direct and indirect exposures deriving from interactions or proximities with DTs and their combined influence on health across the lifespan. This definition emphasizes exposure rather than mere usage, aligning with the broader exposome principle that environmental factors operate cumulatively and dynamically throughout life.

**Figure 1.**
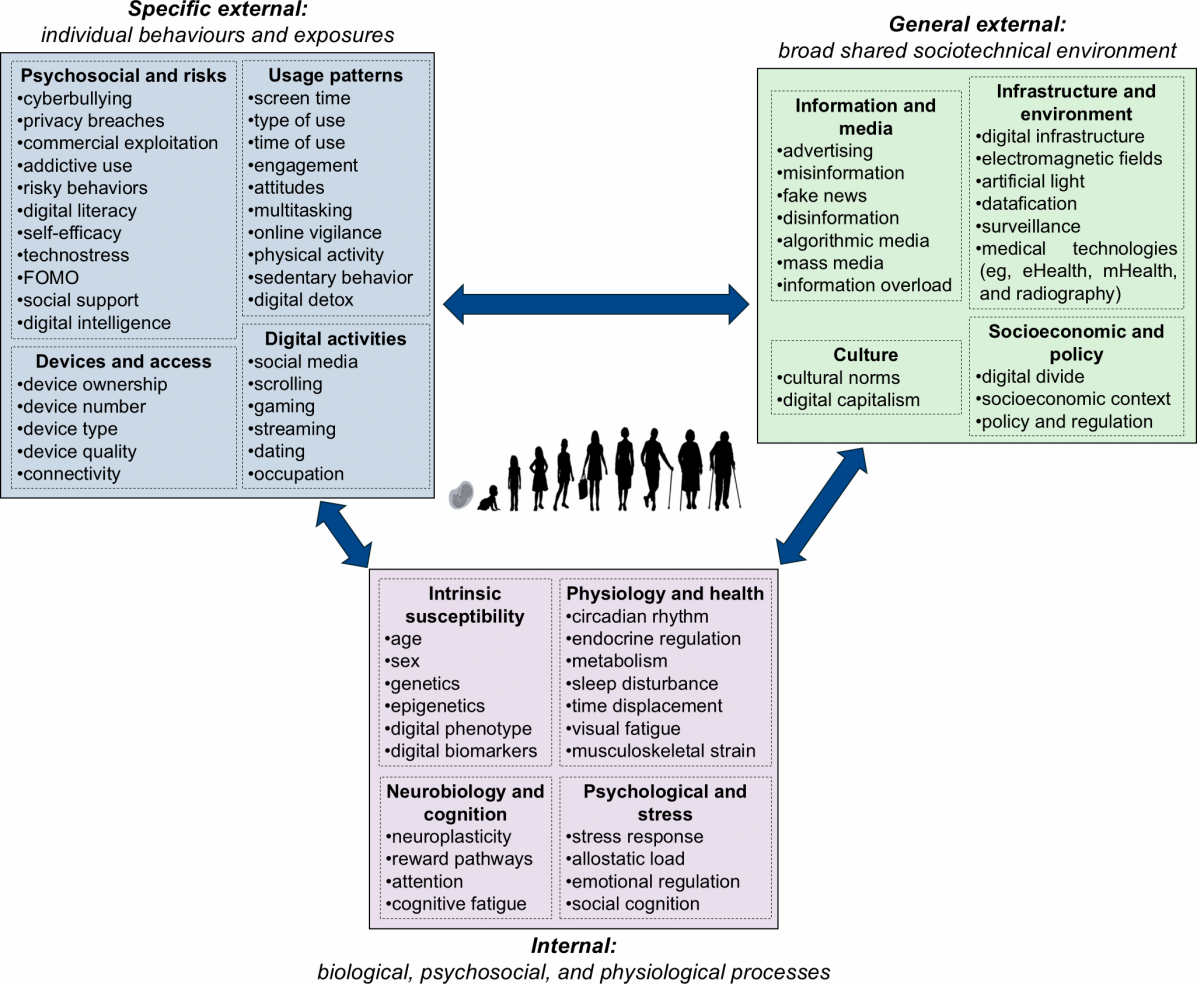
Conceptual framework of the digital component of the exposome. The classical 3-domain exposome structure [15] was adapted for digital technology–related exposures. Bidirectional arrows represent dynamic lifespan interactions, with heightened vulnerability of individuals during developmental windows (eg, childhood and adolescence). FOMO: fear of missing out; mHealth: mobile health.

This redefinition can be positioned within the classical 3-domain structure of the exposome (Figure 1) [15]. Figure 1 synthesizes established exposome theory with emerging scholarship on DT use, digital footprints and tracing [1], digital markers or biomarkers [19,20], and digital phenotyping [2,21,22], resulting in an integrative view of the digital exposome. Each exposome domain is organized into thematic categories. This conceptual representation is informed in interdisciplinary evidence spanning public health, neuroscience, behavioral science, and digital health studies.

The upper part of Figure 1 depicts the 2 external domains of the digital exposome. The general external digital exposome (upper right) refers to population-level, contextual DT-related exposures that operate broadly across societies. These include structural and environmental influences such as misinformation, information overload, blue light exposure, electromagnetic fields, digital advertising ecosystem, digital infrastructure, and algorithmic content curation.

In contrast, the specific external digital exposome (upper left) encompasses individualized and behavioral DT-related exposures shaped by personal usage patterns and access conditions. Examples include screen time, digital literacy, multitasking, cyberbullying experiences, and occupational digital practices. Both external domains involve direct and indirect DT-related exposures. Direct exposures stem from immediate physical or behavioral interactions with DT (eg, screen time, sedentary behavior, and blue light exposure), whereas indirect exposures arise through mediated sociotechnical processes such as algorithmic filtering, addictive digital design, misinformation, or enforced digital disengagement strategies.

The lower part of Figure 1 represents the internal digital exposome domain, which captures biological, physiological, neurocognitive, and psychosocial responses to external digital stimuli. This domain includes processes such as neuroplasticity, stress activation, cognitive fatigue, sleep disturbance, appetite changes, and circadian disruption or metabolic alterations.

The conceptualization assumes dynamic and bidirectional interactions between internal susceptibilities and external DT-related exposures throughout the life course. The 3 domains of the digital exposome interact dynamically across the lifespan and are particularly influential during sensitive developmental stages such as childhood and adolescence, when exposures can have lasting and disproportionate effects [12,23]. For example, adolescence represents a sensitive period in which many young people spend more time on digital platforms (eg, smartphones, tablets, and computers) than with peers or family members [12,24].

## Health Impacts Across the Lifespan

DTs exert complex, bidirectional effects across the lifespan [25]. The digital exposome influences multiple organ systems through physiological and behavioral pathways, including cardiovascular strain from sedentary lifestyles, metabolic dysregulation, and endocrine and immune alterations [2,10,17]. The adverse consequences of DT-related exposures can be broadly categorized into direct and indirect effects. Direct effects arise from the content or functional characteristics of digital media (eg, multitasking, push notifications, and blue light exposure), while indirect effects result from displacement of other essential activities, including sleep, physical exercise, and schoolwork [12].

Mental health and cognitive development represent the most extensively studied domains, particularly since the COVID-19 pandemic [12]. DT use has been associated with depression, anxiety, low self-esteem, addiction, and suicidal ideation [2,10,17], although causal relationships remain difficult to establish [24]. For example, among adolescents, very high daily time spent on digital media (≥6 hours/day), excluding schoolwork, correlates with lower self-esteem, depressive symptoms, and poorer sleep quality [12]. In addition, generative AI technologies may expose children to unsafe or inappropriate content, further compounding risk [11].

Beyond mental health, DTs contribute to a range of other disorders, including ocular conditions [4,10], noise-induced hearing loss [4], musculoskeletal disorders and pain syndromes [4,10,25,26], and obesity [10]. Blue light exposure during daytime may enhance attention, but nighttime exposure disrupts circadian rhythms and is associated with sleep disturbances and other health risks [27]. Prolonged screen use similarly impairs sleep quality, disrupts circadian regulation, and reduces next-day alertness [10,28]. Infodemics during COVID-19 illustrate population-level consequences of digital misinformation that negatively affects disease outbreak management [29,30]. Excessive reliance on DT may also contribute to cognitive decline, a phenomenon often described as digital dementia [3,31]. The digitalization of workplaces has further reshaped occupational settings, improving efficiency in some contexts while simultaneously heightening psychosocial stress and ergonomic risks [25].

Importantly, these effects are unevenly distributed, as illustrated by the digital determinants of health framework [13,14]. Populations with limited digital literacy, inadequate access, or lack of algorithmic transparency face greater exposure risks and fewer benefits, perpetuating the digital divide and reinforcing health inequities [5].

## Barriers to Integration

Integrating the digital exposome into health science presents methodological, practical, and ethical challenges [2,24,32-34]. DT-related exposures are ubiquitous, rapidly evolving, and deeply interwoven with daily life, making them difficult to measure and mitigate.

Current assessments rely predominantly on self-reported measures or indicators such as screen time or digital media use, which correlate only modestly with objective device logs and suffer from low validity [12,24,33-36]. These measures fail to capture content quality, engagement type, multitasking, contextual use (eg, educational vs recreational), or biological correlates [33,34,36]. Ecological momentary assessment offers real-time behavioral data in naturalistic settings through ambulatory techniques such as diaries and physiological recordings [37,38]. By prompting multiple daily self-reports, ecological momentary assessment minimizes recall bias but struggles with longitudinal participant engagement [38].

Screen exposure quantification remains inconsistent. Self-reporting is subjective [39], eye-tracking glasses are invasive (particularly for children) and visually selective [39], and built-in smartphone apps lack cross-device compatibility and user verification [39,40]. A recent study proposed combining egocentric cameras with object detection to track multiscreen exposure patterns [39].

Passive smartphone and wearable sensing holds promise [16,39] but raises complex ethical challenges akin to broader exposome research. Continuous, granular data streams (location tracking, tappigraphy, and physiological signals) are often reidentifiable, creating issues of privacy, informed consent, data sovereignty, equity, governance, and actionability [1,32,41,42]. The General Data Protection Regulation–aligned solutions (eg, data minimization, on-device preprocessing, and deidentification) are essential but leave open questions about longitudinal analysis, secure storage, responsible sharing, and equitable benefit-sharing with disproportionately burdened populations [2,42]. Current normative frameworks remain insufficient for exposome data governance [41].

Traditional cross-sectional designs provide static snapshots, missing within-person variation over time and context [38,43]. Inconsistent DT-related exposure definitions impede study comparability [1,10,17], while SDOH confounding complicates causal inference [13,14]. SDOH are rarely discrete or easily measured, making it difficult to establish the well-defined exposures required for valid causal analysis [44,45]. While some SDOH components (eg, specific socioeconomic status pathways) may be more actionable than commonly recognized in epidemiology [46], causal inference from observational data still requires causal consistency (ie, specifying plausible interventions to shift exposure states) [44]. Decisions about modeling distal SDOH as confounders versus causal variables carry important methodological implications that require explicit justification in exposome research [46]. Robust modeling, confounder management, and interpretable pipelines remain essential to avoid spurious associations [45]. The expotype concept, defined as the vector of exposures accumulated over time, further underscores that clinical integration requires causal validation [46].

## Future Directions

Addressing these challenges requires multidisciplinary collaboration (eg, clinicians, epidemiologists, neuroscientists, psychologists, sociologists, computer scientists, patients, and digital health experts). Priority actions include developing harmonized metrics for DT-related exposures, embedding digital variables into ExWAS [16], and linking digital data with health outcomes [2,21,22].

Strengthening digital literacy through education and clinical training can empower health care professionals to recognize and manage digital risks in practice [4]. Clinical assessments should incorporate patterns of digital usage, behavioral addiction screening, and circadian disruption indicators, supporting personalized interventions such as guided digital detoxification or AI-supported cognitive therapies [8,9].

Large-scale longitudinal studies are urgently needed to clarify how DT-related exposures shape health trajectories across the life course, examining cumulative burden, timing, intensity, and context [23,33,43]. Key questions include the following: At which life stages are DT-related exposures most harmful or beneficial? How do lifelong digital habits interact with aging, chronic disease, and physical and cognitive decline? Which environmental factors influence the adoption, frequency, intensity, and type (eg, problematic) of DT use?

Evidence-based public health guidelines must evolve to reflect the complexity of modern digital environments. Current screen time recommendations remain limited, often overlooking important distinctions between passive use and active, purposeful engagement [10,47,48]. Updated guidance should consider environmental factors, developmental stages, and emerging evidence on both the benefits and risks of DT-related exposure [10,48].

Operationalizing the digital exposome requires objective metrics and validated instruments such as the Media and Technology Usage and Attitudes Scale [49], Cyberbullying Questionnaire [50], problematic internet use scales [51], and eHealth literacy tools [52]. Standardized metrics should include device logs (eg, unlocks, app categories, and late-night use) and contextual self-reports (eg, purpose and weekday or weekend patterns). Behavioral indicators (eg, app switches, concurrent windows, and notification responsiveness) require harmonization across studies. Objective monitoring approaches are becoming increasingly feasible, including in-vivo measures of online behavior (eg, platform-specific time-use data and coded analysis of social media interactions) [43] and smartphone apps that can passively log screen-on duration [36,53], track fine-grained touchscreen interactions (“tappigraphy”) [40], or merge logging with experience sampling [54]. For example, among older adults, analyses of call detail records have been used to explore the relationship between social asymmetry and depressive symptoms, demonstrating how digital trace data may reveal psychosocial vulnerability [55]. Wearable and mobile sensors enable highly individualized, high-resolution exposure and physiological monitoring [16]. Embedding digital variables into ExWAS could facilitate systematic linkage of DT-related exposures with biological and clinical outcomes.

Occupational medicine should quantify “digital task load” (eg, hours of intensive email use, number of online meetings, after-hours connectivity, screen breaks, or multitasking patterns) and relate it to adverse health outcomes such as psychosocial hazards (eg, burnout) and musculoskeletal issues.

Digital markers or biomarkers, defined as objective and quantifiable physiological or behavioral measures derived from digital devices, offer new opportunities to link continuous real-world data with health outcomes [19,20]. Complementing this approach, the digital phenotype refers to the measurable footprint of an individual’s digital behavior, encompassing patterns of interaction, communication, and technology use [1,2,19,21,22]. Smartphone-based digital phenotyping provides scalable, longitudinal assessment that links environmental context and behavioral data with chronic disease trajectories [22], allowing high-resolution profiling of mobility, behavior, and environment [22]. Together, these tools can bridge digital behavior and biological processes, enabling early identification of risk and resilience. Integrating digital markers or biomarkers and phenotypes into exposome research will enhance our ability to monitor, predict, and personalize health trajectories, advancing precision and personalized medicine in the digital age [21,22].

## A Call to Action

The digital revolution is irreversible; however, its potential health consequences remain within our control. The digital exposome represents a defining determinant of contemporary health, encompassing both risks and opportunities. Its systematic integration into exposome science, clinical medicine, digital health, and public health is essential to ensure that digital environments promote rather than compromise well-being. This systematic integration is not merely conceptual but practically achievable through validated instruments [49,50], objective digital logging [53,54], longitudinal phenotyping [22], and harmonized DT-related exposure metrics.

The coming decade must turn awareness into action. Coordinated collaboration among researchers, clinicians, educators, policymakers, and funders can transform the digital exposome from a conceptual frontier into a foundation for healthier societies. By systematically recognizing and addressing DT-related exposures as integral components of the human environment, we can shape a future in which technological progress and public health advance together.

## Abbreviations

AI: artificial intelligence
DT: digital technology
ExWAS: exposome-wide association study
SDOH: social determinants of health
